# Transfer Learning to Detect COVID-19 Automatically from X-Ray Images Using Convolutional Neural Networks

**DOI:** 10.1155/2021/8828404

**Published:** 2021-05-15

**Authors:** Mundher Mohammed Taresh, Ningbo Zhu, Talal Ahmed Ali Ali, Asaad Shakir Hameed, Modhi Lafta Mutar

**Affiliations:** ^1^College of Information Science and Engineering, Hunan University, Changsha 400013, China; ^2^Department of Mathematics, General Directorate of Thi-Qar Education, Ministry of Education, Thi-Qar, Iraq; ^3^Faculty of Information and Communication Technology, Universiti Teknikal Malaysia Melaka, Hang Tuah Jaya, Durian Tunggal, Melaka, Malaysia

## Abstract

The novel coronavirus disease 2019 (COVID-19) is a contagious disease that has caused thousands of deaths and infected millions worldwide. Thus, various technologies that allow for the fast detection of COVID-19 infections with high accuracy can offer healthcare professionals much-needed help. This study is aimed at evaluating the effectiveness of the state-of-the-art pretrained Convolutional Neural Networks (CNNs) on the automatic diagnosis of COVID-19 from chest X-rays (CXRs). The dataset used in the experiments consists of 1200 CXR images from individuals with COVID-19, 1345 CXR images from individuals with viral pneumonia, and 1341 CXR images from healthy individuals. In this paper, the effectiveness of artificial intelligence (AI) in the rapid and precise identification of COVID-19 from CXR images has been explored based on different pretrained deep learning algorithms and fine-tuned to maximise detection accuracy to identify the best algorithms. The results showed that deep learning with X-ray imaging is useful in collecting critical biological markers associated with COVID-19 infections. VGG16 and MobileNet obtained the highest accuracy of 98.28%. However, VGG16 outperformed all other models in COVID-19 detection with an accuracy, F1 score, precision, specificity, and sensitivity of 98.72%, 97.59%, 96.43%, 98.70%, and 98.78%, respectively. The outstanding performance of these pretrained models can significantly improve the speed and accuracy of COVID-19 diagnosis. However, a larger dataset of COVID-19 X-ray images is required for a more accurate and reliable identification of COVID-19 infections when using deep transfer learning. This would be extremely beneficial in this pandemic when the disease burden and the need for preventive measures are in conflict with the currently available resources.

## 1. Introduction

The novel coronavirus disease 2019 (COVID-19) pandemic remains a worldwide concern, threatening to devastate global health. Early detection of infections is one of the first lines of defence against this pandemic, in a bid to reduce the spread of infections [1, 2]. While Reverse Transcription-Polymerase Chain Reaction (RT-PCR) is the current gold standard for disease diagnosis, molecular testing of respiratory tract specimens is also highly recommended, which allows for laboratory confirmation of infections. However, the dramatic proliferation of COVID-19 has resulted in an insufficient number of laboratory kits, creating a significant challenge [3]. Thus, the use of radiological examinations in identifying infections has become increasingly attractive during the COVID-19 outbreak [4].

Although computed tomography (CT) scans have proven to be more effective, the increasing number of patients and the consequent rise in radiological examinations are making it impossible to continuously rely on chest CT scans for each individual patient from diagnosis to discharge. Also, a high reliance on CT scans will impose a significant burden on radiology departments, thus rendering chest X-rays (CXRs) a more feasible option for COVID-19 detection [5]. Although CXRs are deemed less sensitive in diagnosing early-stage pulmonary involvement in COVID-19, it is beneficial to track the gradual development of lung anomalies [6]. Previous studies have observed and identified various radiological manifestations of COVID-19, such as consolidation, reticular interstitial thickening, ground-glass opacities (GGO), pulmonary nodules, and pleural effusion [7, 8].

With the rapid global spread of COVID-19, researchers have begun using state-of-the-art deep learning techniques for the automated detection of COVID-19 within patients. The onerousness of obtaining COVID-19 data in its initial stages has forced researchers to create their own model using pretrained networks [9–22]. However, the bulk of these experiments used a limited dataset comprising just a few COVID-19 samples. This renders the stated results in these studies are difficult to generalize and does not ensure the reported output would be retained when these models are evaluated on a larger dataset. Therefore, the transfer learning approach for detecting COVID-19 X-ray images must be verified on a large dataset. In addition to the fact that the combination of healthy and pneumonia cases is considered inappropriate where the model would attempt to disregard the intraclass variation between these two classes, the accuracy obtained in this way is not an accurate measure [23].

Deep learning has been shown to play an important role in distinguishing between viral and bacterial pneumonia [24–26] and diagnosing the most common thoracic diseases [27–29]. Moreover, the challenge is to develop an algorithm capable of identifying a patient with COVID-19. Nevertheless, this task remains challenging as COVID-19 can share similar radiographic features with other types of pneumonia. In [10], the authors mentioned the poor performance of MobileNet in distinguishing cases of COVID-19 from other pneumonia cases when the training dataset included only bacterial pneumonia cases. We thus attempt to distinguish COVID-19 from viral pneumonia (not bacterial pneumonia) by aiming to rapidly detect clusters of COVID-19 caused by a novel virus. Furthermore, the COVID-19 versus non-COVID classification is a severe imbalance problem regarding the number of COVID-19 versus non-COVID-19 samples due to the difficulty of obtaining an adequate number of positive COVID-19 samples.

This paper is aimed at reducing both the false-positive and false-negative rate as much as possible. The number of frozen layers has been shown to affect the recognition capability of pretrained models [30]. However, no work has been carried out to investigate the performance of the popular pretrained models with different number of frozen layers, and previous works have not comprehensively considered comparative analysis of these models' performances in COVID-19 diagnosis. Therefore, it is sensible to tune the frozen layers to utilize the full potential of pretrained models in order to improve COVID-19 recognition capability. With this goal in mind, eight popular pretrained deep learning networks were compared in terms of various performance metrics, each with different numbers of frozen layers. This enabled the identification of the best framework in the extraction of COVID-19 manifestations. Thereby, our work differs from the prior proposals [10, 13, 21, 22] in that the proposed model is not only evaluation-based but also COVID-19-specific. As such, this work could potentially help researchers to identify the best-practice CNN descriptor, i.e., the appropriate deep CNNs with proper layers and convolutional blocks to extract the features of COVID-19. The contributions of this work are summarized as follows.

- The development of a state-of-the-art automated COVID-19 diagnosis framework based on a pretrained deep learning model is proposed. The developed model exploits the VGG16 CNN with 18 frozen layers to detect COVID-19 patients using CXR images
- Provides insight into the performance of each of the eight well-known pretrained models with different frameworks arising by adjusting the number of the frozen layers and identifying the number of trainable convolutional blocks
- A comparative study of eight well-known pretrained CNN models, with different frameworks, is conducted through many experiments on a large dataset consisting of CXRs from individuals with COVID-19, demonstrating the superiority of the proposed framework in (i) in terms of achieving the least number of false positives and false negatives
- Identifying the best performance of each model and then evaluated it on the test set again to make sure the model is more generalizable

This paper is organized as follows.  Section 2 describes the methods used in this study. The quantitative results and discussion are presented in Section 3, while Section 4 illustrates the approach taken for comparison with previous studies. Finally, the conclusion of this study is presented in Section 5.

## 2. Materials and Methodology

**Figure 1:**
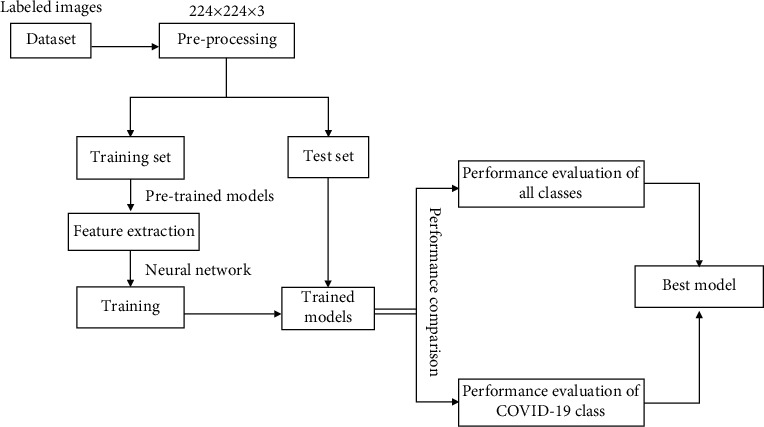
Block diagram of the proposed method.

In this section, we briefly describe the approach used to achieve the objectives of the study. The diagram of the proposed method is represented in Figure 1.

### 2.1. Dataset and Input Preprocessing

**Figure 2:**
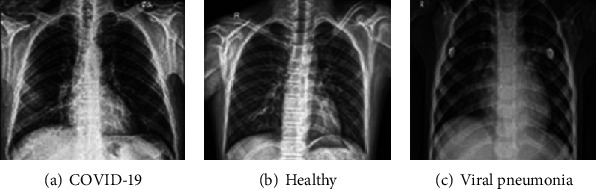
Samples of X-ray images used in this study.

In this work, the chest X-ray image dataset was downloaded from https://www.kaggle.com/tawsifurrahman/covid19-radiography-database, 17 December 2020, which was prepared by the authors of [21], who undertook the tedious task of collecting and indexing the X-ray images. This dataset consists of CXRs from 1200 individuals with COVID-19, 1341 CXRs from healthy individuals, and 1345 CXRs from individuals with other types of viral pneumonia. All the images are in the Portable Network Graphics (PNG) file format, and with a resolution of either 1024-by-1024 pixels or 256-by-256 pixels. It must be noted that the dataset is divided into 3575 training and 311 test images, as outlined in Table 1. In the training phase, the dataset was prepared and verified as reliable by reviewing it with chest specialists. In addition, cases of viral pneumonia should be free from any instances of COVID-19. Before passing the images into a pretrained model for feature extraction, we resized all images to a size of 224 × 224 × 3 pixels. All images were normalized according to the pretrained model standards.  Figure 2 shows examples of CXR images within the training set that were used in this study.

### 2.2. Pretrained Model and Sequential Model

In this study, the transfer learning technique was applied using ImageNet data to resolve the problems of inadequate data and preparation time. The weights trained on ImageNet were downloaded for each model. The feature maps were treated as input size in the applied layers training process. Moreover, for fine-tuning, a brief description of the CNNs employed for automatic detection was created.  Table 2 shows the parameters of the applied CNNs in terms of classification function and transfer learning criteria. The fine-tuning parameters were determined after several experiments. The frozen layer parameter refers to the number of untrainable layers starting from the bottom of the CNN, which is good because their weights are not expected to change during model training. The bottleneck features parameter refers to the last feature map that was flattened to feed a fully connected deep neural network classifier during the pretraining.

**Figure 3:**
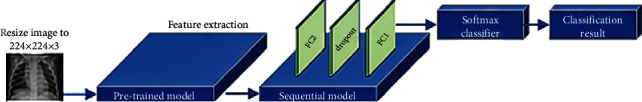
Outline of the method.

The other layers that were closer to the output features were trained to extract more information from the later convolution layers. We added three more layers to the top of each model, namely, a fully connected layer (FC2) with the output of 512, a dropout layer, and another fully connected layer (FC1) with a softmax classifier as depicted in Figure 3. A dropout layer [31] was added to prevent overlapping [32], passing with 0.5 for each neural network used in this study. The network was trained with a softmax classifier for 15 epochs using an RMSprop optimizer [33], with a learning rate of 0.00001 and a batch size of 32. Simultaneously, the sequential model used as a classifier with the last three layers includes the softmax classifier. The frozen layer and bottleneck features parameters are shown in Table 2.

### 2.3. Performance Metrics

This subsection describes the evaluation of the performance of different deep learning models for classifying the CXR images. The trained models were validated using tenfold cross-validation, and the performance metrics derived from the confusion matrix were used for experimental analyses. The confusion matrix provides a guideline to the four outcomes of false negative (FN), false positive (FP), true negative (TN), and true positive (TP). The presence of both FNs and FPs could affect medical decisions negatively. An FP result is produced when an individual is inaccurately assigned to a class, such as when a healthy individual is incorrectly categorized as a COVID-19 patient. An FN occurs when an individual who is supposed to fall into a given class is instead excluded from this group. The performance of the different networks was evaluated on the test set by computing the macroaverage of accuracy (Acc), *F*1 score, precision (PPV), specificity (Spc), sensitivity (Sen), and Matthew Correlation Coefficient (MCC) [34] as quantitative evaluation indices. These are defined as

(1) $$\begin{array}{c} \text{Accuracy}{\left(\text{Acc}\right)}_{i}=\frac{{\text{TP}}_{i}+\text{T}{\text{N}}_{i}}{{\text{TP}}_{i}+{\text{FP}}_{i}+{\text{TN}}_{i}+{\text{FN}}_{i}}, \end{array}$$

(2) $$\begin{array}{c} F1\text{ scor}{\text{e}}_{i}=2\times \frac{\;{\text{PPV}}_{i}\times {\text{Sen}}_{i}}{{\text{PPV}}_{i}+{\text{Sen}}_{i}}, \end{array}$$

(3) $$\begin{array}{c} \text{Precision}{\left(\text{PPV}\right)}_{i}=\frac{\;{\text{TP}}_{i}}{{\text{TP}}_{i}+{\text{FP}}_{i}}, \end{array}$$

(4) $$\begin{array}{c} \text{Specificity}{\left(\text{Spc}\right)}_{i}=\frac{{\text{TN}}_{i}}{{\text{FP}}_{i}+{\text{TN}}_{i}}, \end{array}$$

(5) $$\begin{array}{c} \text{Sensitivity}{\left(\text{Sen}\right)}_{i}=\frac{{\text{TP}}_{i}}{{\text{TP}}_{i}+{\text{FN}}_{i}}, \end{array}$$

(6) $$\begin{array}{c} {\text{MCC}}_{i}=\frac{\;\left({\text{TP}}_{i}\times {\text{TN}}_{i}\right)-\left({\text{FP}}_{i}\times {\text{FN}}_{i}\right)}{\sqrt{\left({\text{TP}}_{i}+{\text{FP}}_{i}\right)\left({\text{TP}}_{i}+{\text{FN}}_{i}\right)\left({\text{TN}}_{i}+{\text{FP}}_{i}\right)\left({\text{TN}}_{i}+{\text{FN}}_{i}\right)}}, \end{array}$$

where *i* refers to the class of COVID-19, healthy, and viral pneumonia.

Due to the imbalanced data, it is difficult to determine which classifier performs better in the detection of COVID-19 infections. Misdiagnosis can potentially lead to severe consequences, especially where the COVID-19 cases are concerned. Therefore, it is necessary to calculate the parameters of the COVID-19 class.

## 3. Results and Discussion

In this paper, the experiments were implemented via utilizing Python programming language. All experiments were conducted with a Tesla K80 GPU graphics card on Google Collaboratory with an Intel© i7-core @3.6 GHz processor and a 16 GB RAM on 64-bit Windows 10 operating system.  Table 3 illustrates the comparative computational times of the tested deep learning image classifiers in seconds. The running time of all deep learning models was relatively short, ranging from 363.0 to 2170.0 seconds.

**Figure 4:**
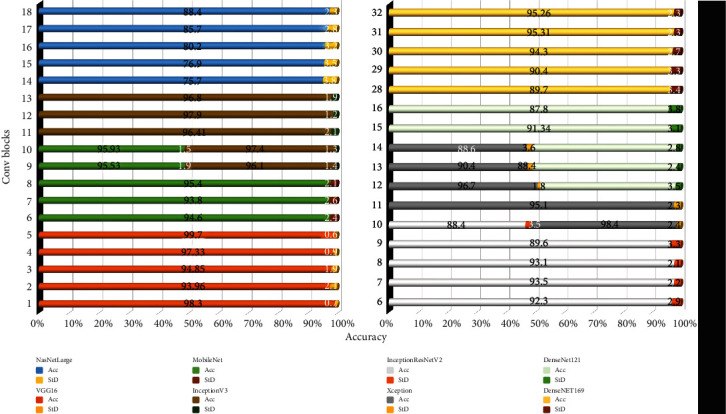
Accuracy versus convolutional blocks and standard deviation of each experiment.

Beforehand, we conducted many experiments on a large chest X-ray dataset of COVID-19 samples fed into eight well-known pretrained CNN models, namely, InceptionV3, Xception, InceptionResNetV2, MobileNet, VGG16, DenseNet169, NasNetLarge, and DenseNet121, each with different selections of frozen layers and number of trainable convolution blocks. All models were trained via 10-fold cross-validation. Furthermore, we chose the best performance of each model separately and finally evaluated each model on test set.  Figure 4 indicates how the number of fine-tuned convolutional blocks influenced the classification performance where the mean classification accuracy and standard deviation (StD) are shown as well. For all pretrained CNN models InceptionV3, Xception, InceptionResNetV2, MobileNet, VGG16, DenseNet169, NasNetLarge, and DenseNet121, the best performance was obtained when we trained 12, 12, 7, 10, 5, 31, 18, and 13 convolution blocks, respectively.

**Figure 5:**
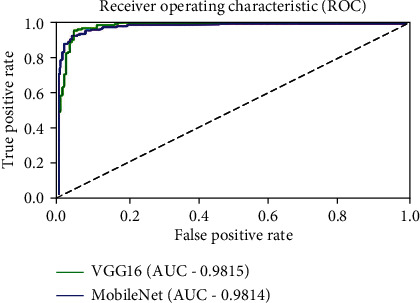
Comparison of ROC curve of VGG16 and MobileNet.

Table 4 summarizes the comparative performance for each of the different pretrained CNN models. It is apparent from Table 4 that all the evaluated pretrained models performed well in classifying COVID-19, healthy, and viral pneumonia CXR images. While there was only a marginal difference between the results of the various CNNs, VGG16 and MobileNet obtained the highest accuracy of 98.29%. However, VGG16 outperformed MobileNet in the aspects of *F*1 score, PPV, and Sen while failing MCC, Spc, and testing time. The receiver operating characteristic (ROC) curve for VGG16 and MobileNet is shown in Figure 5.

**Figure 6:**
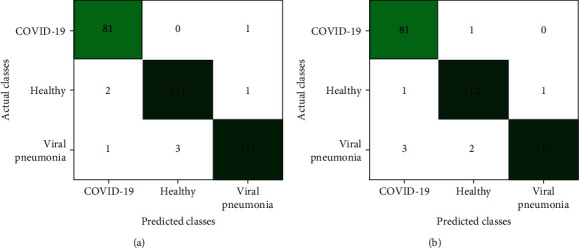
Confusion matrix of (a) VGG16 and (b) MobileNet.

The confusion matrices of VGG16 and MobileNet show consistency between the predicted and actual results, thus indicating better performance as demonstrated in Figure 6. It is clear from Figure 6(a) that only one COVID-19 CXR image was misclassified as a viral pneumonia CXR image. None of the other COVID-19 CXR images were misclassified as healthy images. Two healthy and viral pneumonia CXR images were misclassified as COVID-19 by the VGG16 model. In contrast, Figure 6(b) shows the results from the MobileNet model, where only one COVID-19 CXR image was misclassified as healthy. None of the other COVID-19 CXR images were misclassified as viral pneumonia CXR images. One healthy and three viral pneumonia images were also misclassified as COVID-19 CXR images. In general, only one healthy CXR image was misclassified as a viral pneumonia CXR image in both models. COVID-19 images being misclassified as healthy CXR images has more harmful consequences than it being misclassified as another disease class (i.e., viral pneumonia). In contrast, healthy images that are misclassified as viral pneumonia images have less severe consequences as opposed to when it is misclassified as COVID-19. It can be said that VGG16 does not confuse COVID-19 and healthy images, but it confuses COVID-19 with viral pneumonia. In contrast, the MobileNet model confuses healthy and COVID-19 images, but does not confuse COVID-19 with viral pneumonia images. However, the high precision and *F*1 score show that the network still performs excellently in the reliable classification of most images, which is of utmost importance, as the computer-aided system (CAD) should not classify any COVID-19 patients as healthy or vice versa.

The performance of each CNN concerning COVID-19 cases is presented in Table 5. In terms of COVID-19 detection accuracy, Table 6 shows that VGG16, MobileNet, InceptionV3, and DenseNet169 models outperformed the rest of the models.

**Figure 7:**
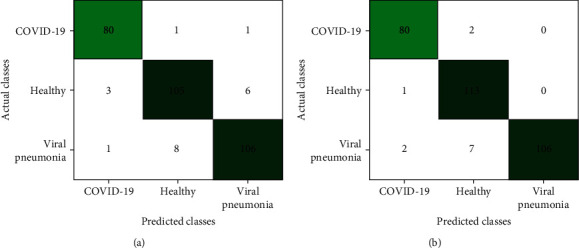
Confusion matrix of (a) DenseNet169 and (b) InceptionV3.

Moreover, Figure 7 shows that DenseNet169 and InceptionV3 have more FN cases. The most optimal models are those with the lowest number of FN when concerning a specific disease. After all, an FN scenario will contribute to the misguided belief that the patient is not infected, which may eventually contribute towards the propagation of infections among the healthy population. Therefore, out of these four models, VGG16 excelled in achieving values up to 98.72%, 97.59%, 96.43%, 98.69%, and 98.78% for Acc, *F*1 score, PPV, Spc, and Sen, respectively.

## 4. Comparison with State-of-the-Art Methods

AI techniques regarding image classification can assist in the early diagnosis of diseases. When AI techniques are incorporated, CNN methods are able to achieve better results as compared to other classification methods [35, 36]. Although there are a large number of COVID-19 patients worldwide, there is only a small number of publicly available CXR images scattered online. However, several recent works have reported promising results when using the transfer learning approach to detect COVID-19 CXR images from a small dataset. To evaluate the proposed CNN model, the general performance comparison of our study with the state-of-the-art methods is shown in this section. In the model evaluations, these related studies depend on multiclass classification of CXR images with various AI techniques.

Apostolopoulos and Mpesiana [10] established a deep learning model using 224 confirmed COVID-19 images, and their model achieved a performance rate of 98.75% and 93.48% when using two and three classes, respectively. Wang et al. [37] proposed a deep COVID-19 detection model (COVID-Net) that achieved 93.3% accuracy in the classification of three categories (healthy, non-COVID-19 pneumonia, and COVID-19). A CNN model called DarkNet was proposed by [13] and achieved a performance rate of 98.08% and 87.02% for two and three classes, respectively. Khan et al. [22] proposed a CoroNet model to classify CXR images that managed to achieve an accuracy of 99% and 95% for 2-class and 3-class classification tasks, respectively, on a dataset containing 310 COVID-19, 657 pneumonia, and 284 healthy CXR images. Toraman et al. [38] proposed a convolutional CapsNet for detecting COVID-19 by using CXRs with capsule networks. Their proposed method achieved an accuracy of 97.24% and 84.22% for binary and multiclasses, respectively. The transfer learning technique was used in [21] with image augmentation to train and validate several pretrained deep CNNs. Their proposed method achieved an accuracy of 97.94% for DenseNet201 with image augmentation and 97.74% for CheXNet without image augmentation.

Our best-performing CNN was also tested on another dataset prepared by Chowdhury et al. [21] to verify its generalizability and robustness further. The dataset contains 1341 healthy, 1345 non-COVID viral pneumonia, and 423 COVID-19 CXR images. All the images used are from the same source as the dataset used in this study. Our method obtained an accuracy of over 96% on this dataset after training, as outlined in Table 6.

Table 7 shows the comparison between the method proposed in this study and the current classification method of COVID-19, non-COVID viral pneumonia, and healthy CXR images. Each index in Table 7 was directly taken from their original source papers, in view of the fact that their datasets were either ever-growing or not publicly available. Meanwhile, as different models use different datasets, comparisons between different researches are made more difficult. Nevertheless, our method was characterized by a fast and smooth execution, though it was trained on a relatively large dataset. The results demonstrate that the approach suggested by our research generally worked better. Performance was improved by conducting several experiments on the CXR dataset to identify which layer extracted the best features for the detection of COVID-19, to obtain the best results.

Although Table 7 shows that a better performance value was achieved in [21], our result remains acceptable due to the fact that image augmentation was performed in [21] to achieve this value, while 95.19% was obtained without image augmentation.

The promising deep learning models used for the detection of COVID-19 from radiography images indicate that deep learning likely still has untapped potential and can possibly play a more significant role in fighting this pandemic. There is definitely still room for improvement, through other processes such as increasing the number of images and implementing preprocessing techniques (i.e., data augmentation and/or image enhancement).

## 5. Conclusions

In this paper, different pretrained deep learning networks were applied to identify the best deep learning technique in terms of extracting the various COVID-19 manifestations. Many experiments were conducted using the CXR dataset to recognize which layer is able to extract the best features to obtain the best performance. It was observed that deep networks performed well in classifying COVID-19, healthy, and viral pneumonia CXR images—especially the VGG16 and MobileNet networks, which surpassed other networks in all metrics. The results also showed the superiority of VGG16, MobileNet, DenseNet169, and InceptionV3 in identifying COVID-19 CXR images with a high sensitivity and accuracy. However, excellence in high performance remained besides VGG16 with high precision. The classification accuracy, *F*1 score, precision, specificity, and sensitivity of COVID-19 were 98.72%, 97.59%, 96.43%, 98.7%, and 98.78%, respectively. This study demonstrated that deep learning with X-ray imaging might be able to extract significant biological markers that are related to the COVID-19 disease.

However, this study does have its shortcomings. In particular, a more detailed analysis requires a larger amount of patient data, especially COVID-19 data. After all, effective deep learning models are usually trained on more than a million images, a number that is difficult to obtain in the medical domain. Besides, there is a possibility that training deep neural networks on limited dataset results in overfitting and hinders its generalization. Visual ablation studies can be performed along with deep transfer learning, which will significantly improve the detection of COVID-19 manifestations in the CXR images.

## Figures and Tables

**Table 1:** Summarized dataset for training and testing.

Data	COVID-19	Healthy	Viral pneumonia	Total images
Train	820	1140	1150	3575
Test	82	114	115	311

**Table 2:** The parameters of CNNs for transfer learning.

Classifier	Frozen layers	Bottleneck features
InceptionV3	230	27648
Xception	116	142688
InceptionResNetV2	779	38400
MobileNet	66	100352
VGG16	18	25088
DenseNet169	575	6272
NasNetLarge	858	32928
DenseNet121	403	6272

**Table 3:** Computational times of all tested CNNs on a GPU (s).

Classifier	Training time	Testing time
InceptionV3	280	83
Xception	660	135
InceptionResNetV2	980	389
MobileNet	320	54
VGG16	480	138
DenseNet169	799	332
NasNetLarge	1660	510
DenseNet121	740	249

**Table 4:** Parameters of different classification models for all classes with the best value in bold (%).

Classifier	Acc	*F*1	MCC	PPV	Spc	Sen
InceptionV3	97.43	96.22	94.26	96.34	98.04	96.29
Xception	97.86	96.64	95.21	96.54	98.45	96.87
InceptionResNetV2	93.00	90.70	86.38	91.27	95.37	90.36
MobileNet	**98.29**	**97.39**	**96.13**	**97.26**	**98.74**	**97.56**
VGG16	**98.29**	**97.44**	**96.11**	**97.34**	**98.72**	**97.57**
DenseNet169	95.71	93.82	90.26	93.72	96.70	93.95
NasNetLarge	97.00	95.22	93.20	95.25	97.77	95.24
DenseNet121	95.93	93.60	90.75	94.00	96.87	93.32

**Table 5:** Parameters of different CNNs for COVID-19 with the best value in bold (%).

Classifier	Acc	*F*1	PPV	Spc	Sen
InceptionV3	98.39	96.97	96.39	98.69	97.56
Xception	97.43	95.24	93.02	97.38	97.56
InceptionResNetV2	94.21	88.46	93.24	97.82	84.15
MobileNet	98.39	97.01	95.29	98.25	98.78
VGG16	**98.71**	**97.59**	**96.43**	**98.69**	**98.78**
DenseNet169	98.07	96.39	95.24	98.25	97.56
NasNetLarge	96.14	92.68	92.68	97.38	92.68
DenseNet121	95.50	91.14	94.73	98.25	87.81

**Table 6:** Performance of the best CNN on dataset prepared by [21] (%).

	Acc	*F*1	PPV	Spc	Sen
COVID-19	97.43	95.24	93.02	97.38	97.56
Healthy	96.77	95.76	92.62	95.43	99.12
Viral pneumonia	96.14	95.76	100	100	89.56
Overall	96.79	95.17	95.22	97.60	95.42

**Table 7:** General comparison of the best CNN obtained with state-of-the-art method.

Study	Method used	Database size	Acc%
[10]	VGG-19	224 COVID-19, 504 healthy, and 700 pneumonia	93.48
[10]	MobileNet v2	224 COVID-19, 504 healthy, and 700 pneumonia	94.72
[21]	DenseNet201	423 COVID-19, 1341 healthy, and 1345 viral pneumonia	97.94
[38]	CapsNet	231 COVID-19, 1050 healthy, and 1050 pneumonia	84.22
[37]	COVID-Net	358 COVID-19, 8066 healthy, and 5538 pneumonia	93.3
[13]	DarkCOVIDNet	157 COVID-19, 500 healthy, and 500 pneumonia	87.02
[22]	CoroNet	157 COVID-19, 500 healthy, and 500 pneumonia [13]	90.21
[22]	CoroNet	284 COVID-19, 310 healthy, and 657 pneumonia	95
Our work	VGG16	423 COVID-19, 1341 healthy, and 1345 viral pneumonia [21]	96.79
		1200 COVID-19, 1341 healthy, and 1345 viral pneumonia	98.29

## Data Availability

All datasets used in the experiments were obtained from J. C. Monteral (2020) COVID Chest X-ray Database (https://github.com/ieee8023/covid-chestxray-dataset) and COVID-19 Radiography Database (https://www.kaggle.com/tawsifurrahman/covid19-radiography-database).
